# Effect of Potassium Concentration on Triplex Stability under Molecular Crowding Conditions

**DOI:** 10.3390/molecules25020387

**Published:** 2020-01-17

**Authors:** Ye Teng, Hisae Tateishi-Karimata, Tatsuya Ohyama, Naoki Sugimoto

**Affiliations:** 1 Frontier Institute for Biomolecular Engineering Research (FIBER), Konan University, 7-1-20 Minatojima-Minamimachi, Chuo-ku, Kobe 650-0047, Japan; yteng28@163.com (Y.T.); tateishi@konan-u.ac.jp (H.T.-K.); t-ohyama@konan-u.ac.jp (T.O.); 2 School of Pharmacy, Changchun University of Chinese Medicine, 1035 Boshuo Road, Changchun 130117, China; 3 Graduate School of Frontiers of Innovative Research in Science and Technology (FIRST), Konan University, 7-1-20 Minatojima-Minamimachi, Chuo-ku, Kobe 650-0047, Japan

**Keywords:** molecular crowding, triplex stability, nucleic acid

## Abstract

The properties of non-canonical DNA structures, like G-quadruplexes and triplexes, change under cell-mimicking molecular crowding conditions relative to dilute aqueous solutions. The analysis of environmental effects on their stability is crucial since they play important roles in gene expression and regulation. In this study, three intramolecular and intermolecular triplex-forming sequences of different C^+^_*_G-C triplet content (_*_: Hoogsteen base pair; - : Watson–Crick base pair) were designed and their stability measured in the absence and presence of a crowding agent with different K^+^ concentrations. In dilute solution, the stability of the triplexes was reduced by decreasing the concentration of KCl. This reduction became smaller as the number of C^+^_*_G-C triplets increased. Under molecular crowding conditions, Watson–Crick base pairs and Hoogsteen base pairs were destabilized and stabilized, respectively. Interestingly, with lower KCl concentrations (≤1 M), the destabilization of the triplexes due to reduction of KCl concentration was significantly smaller than in dilute solutions. In addition, the C^+^_*_G-C content had greater influence on triplex stability under molecular crowding conditions. Our work provides quantitative information about the effects of K^+^ concentration on triplex stability under molecular crowding conditions and should further our understanding of the function and regulation of triplexes in bioprocesses.

## 1. Introduction

The structure and stability of nucleic acids are important not only for the storage of genetic information but also for the regulation of various bioprocesses such as gene transcription and translation. Non-canonical structures, including triplex, G-quadruplex, i-motif, and others, are considered to play crucial roles in gene expression and regulation [1,2,3], although they are much less abundant than the typical right-handed B-form helix [1]. For example, our group has demonstrated that the formation of non-canonical structures in the template strand caused transcription to pause, slip, and arrest [4]. The formation of a triplex with duplex binding site has also been reported to repress the transcription of c-*Myc* [5]. Obtaining more information about the stability of non-canonical structures such as triplex is necessary in order to further our understanding of gene expression and regulation.

The stability of non-canonical structures is largely dictated by the surrounding environment. As the cells’ surroundings are crowded due to the presence of an abundance of biomolecules, including amino acids, proteins, sugars, lipids and so on, the crowded conditions result in significant effects on the folded structure and stability of nucleic acids when compared with those in dilute conditions [6,7,8]. Our group has previously reported that Hoogsteen base pairs are stabilized while Watson–Crick base pairs are destabilized under molecular crowding conditions [9]. Cations also play important roles in the regulation of bioprocesses by stabilizing the structure of nucleic acids [10]. It is well known that K^+^ stabilizes the formation of G-quadruplex structures [11]. It has also been reported that the formation of the intermolecular purine-purine-pyrimidine triplex was inhibited by the presence of K^+^ [12]. Under molecular crowding conditions, due to the effects of excluded volume and variations in water activity and dielectric constant, the interaction between K^+^ and nucleic acids might also change [6], which will provide significant information to advance our understanding of potassium effects on bioprocesses.

In this work, the effect of K^+^ on the stability of triplex structures was characterized. High concentrations of polyethylene glycol of an average molecular weight of 200 (PEG 200) was added to an aqueous solution as a crowding agent to mimic molecular crowding conditions in cells. Three intramolecular and three intermolecular triplex-forming sequences of differing C^+^_*_G-C content were designed, and their stability was measured in the absence and presence of the crowding agent with different concentrations of K^+^.

## 2. Results

### 2.1. The Structure and Stability of Intramolecular and Intermolecular Triplexes

**Figure 1:**
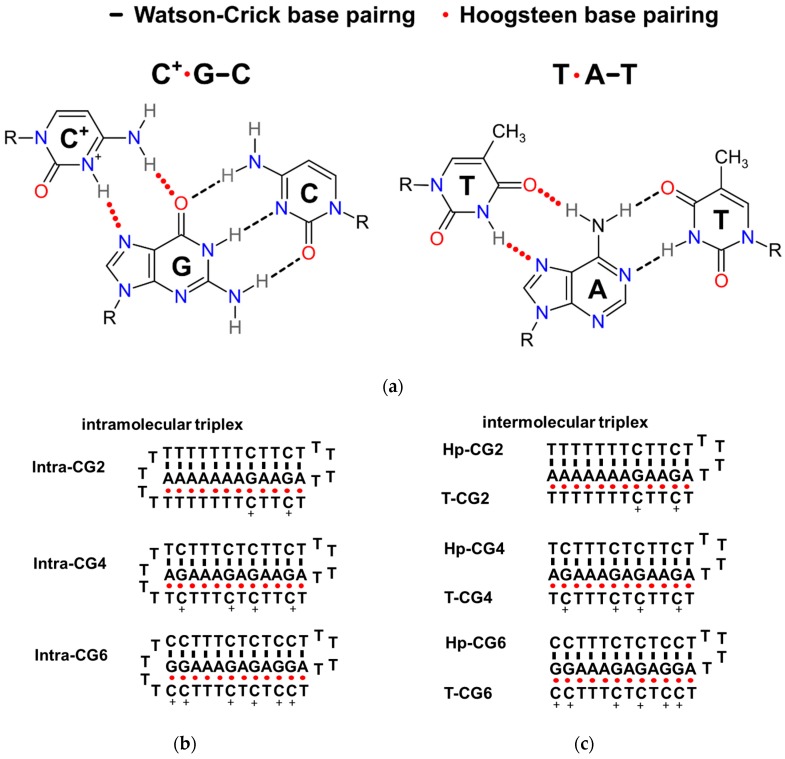
(**a**) The hydrogen bonding patterns of Watson–Crick base pairs and Hoogsteen base pairs in the formation of C^+^_*_G-C and T_*_ A-T. (**b**) The possible formations of intramolecular triplexes in the sequences used in this work. (**c**) The possible formations of intermolecular triplexes in the sequences used in this work.

**Figure 2:**
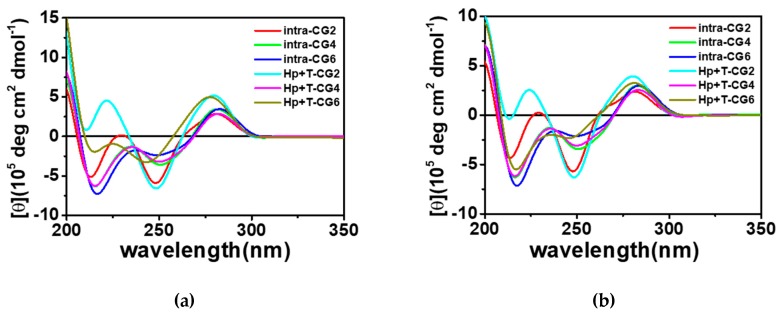
Circular dichroism (CD) spectra of Intra-CG2, Intra-CG4, Intra-CG6, Hp-CG2+T-CG2, Hp-CG4+T-CG4, and Hp-CG6+T-CG6 in a buffer containing 50 mM 2-morpholino-ethanesulfonic acid (MES) and 0.1 M KCl at pH 6.0 in the absence (**a**) and presence (**b**) of PEG 200. The total DNA concentration was 20 μM.

In weakly acidic conditions, not only T*A-T (* denotes Hoogsteen base pair; - denotes Watson-Crick base pair), but also C^+^_*_G-C can form a triplet as shown schematically in Figure 1a. Considering the different hydrogen bonds in T_*_A-T and C^+^_*_G-C, triplexes containing 12 triplets with different ratios of T_*_A-T and C^+^_*_G-C were designed that were based on our previously reported triplex forming sequences [9,13]. The detailed sequences are listed in Table 1. Long continuous C/G sequences were not used in the sequence design to avoid the possible formation of G-quadruplex and i-motif structures. Both intramolecular and intermolecular triplex structures were considered in this study. The possible folding of intramolecular triplexes (Intra-CG2, Intra-CG4, and Intra-CG6), and intermolecular triplexes (Hp-CG2+T-CG2, Hp-CG4+T-CG4, and Hp-CG6+T-CG6) are shown in Figure 1b,c, respectively. We added 40 wt% of PEG 200 as a crowding agent to mimic the cellular environment. The formation of triplexes was further confirmed by circular dichroism (CD) spectroscopy. As shown in Figure 2, in the absence (Figure 2a) and presence (Figure 2b) of PEG 200, the CD spectra had similar results. The negative peak at 240–250 nm and the positive peak at 260–280 nm indicated Watson-Crick base pair in a double helix [14], and the negative peak at 210–220 nm indicated Hoogsteen base pair in a parallel duplex [9]. These results confirmed the triplex formation of all triplex-forming sequences in our experiment. In addition, none of them showed the characteristic G-quadruplex features, including a negative peak at 240 nm and a positive peak at 260 nm, or a negative peak at 260 nm and a positive peak at 290 nm. The results indicated that no large amounts of G-quadruplexes were observed under the condition of 50 mM 2-morpholino-ethanesulfonic acid (MES) (pH 6.0) and 0.1 M KCl in the absence and presence of 40 wt% of PEG 200.

**Figure 3:**
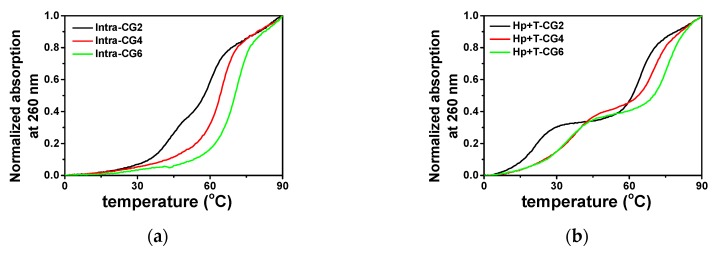
Normalized UV melting curves at 260 nm of (**a**) intramolecular triplexes and (**b**) intermolecular triplexes in a buffer containing 50 mM MES and 0.1 M KCl at pH 6.0 in the absence of PEG 200.

The stability of triplexes was characterized via absorption at 260 nm in a melting process with a heating rate of 0.5 °C·min^−1^. The normalized melting curves in the absence of K^+^ and the crowding agent are shown in Figure 3a,b. The melting curves of the designed intramolecular and intermolecular triplexes showed significantly different features. For the intramolecular triplexes Intra-CG2, Intra-CG4, and Intra-CG6, in a buffer containing 0.1 M KCl and 50 mM MES at pH 6.0, Intra-CG2 showed two close stages in its melting curve, while the melting curves of Intra-CG4 and Intra-CG6 had only one melting stage (Figure 3a). For Intra-CG2, the melting temperatures of Hoogsteen base pairs and Watson-Crick base pairs were 46.5 °C and 59.5 °C, respectively. On the other hand, Intra-CG4 and Intra-CG6 results indicated that the unfolding of Watson-Crick base pairs and Hoogsteen base pairs in those triplexes happened at almost the same time and could not be clearly distinguished by the melting curves. The melting temperatures of Intra-CG4 and Intra-CG6 were 64.1 and 70.2 °C, respectively. The melting curves at 295 nm are correlated to the dissociation of Hoogsteen base pairs. The melting curves at 295 nm can further help us to confirm the dissociation of triplex and distinguish the melting stages of Hoogsteen base pairs from the mixed melting curves at 260 nm for dissociation of Watson-Crick and Hoogsteen base pairs. Thus, dissociation of all Hoogsteen base pairs in the triplexes were further confirmed by the melting curves at 295 nm as shown in Figure S1.

However, as shown in Figure 3b, two melting transitions were observed in all three intermolecular triplexes Hp-CG2+T-CG2, Hp-CG4+T-CG4, and Hp-CG6+T-CG6. The lower melting curve was the melting stage of Hoogsteen base pairs, which was further confirmed by the melting curve at 295 nm (Figure S1). The calculated *T*_m_ values of Hoogsteen base pairs in Hp-CG2+T-CG2, Hp-CG4+T-CG4, and Hp-CG6+T-CG6 were 20.5, 34.0, and 33.5 °C, respectively (Figure 3). The higher melting curve indicated the unwinding of Watson-Crick base pairs, which were 65.5, 71.5, and 76.3 °C, respectively (Figure 3). These results suggest that the melting of Watson-Crick base pairs and Hoogsteen base pairs happened separately, and the single T-strands dissociated more easily from the triplex structure in the absence of K^+^ and crowding agents. Both intramolecular and intermolecular triplexes showed that a higher C^+^_*_G-C content resulted in higher *T*_m_ because of the additional hydrogen bond in C^+^_*_G-C compared with T_*_A-T.

**Figure 4:**
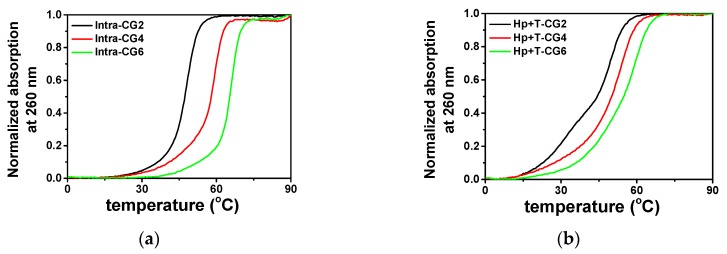
Normalized UV melting curves of (**a**) intramolecular triplexes and (**b**) intermolecular triplexes at 260 nm in the buffer containing 50 mM MES, 0.1 M KCl, and 40 wt% PEG 200 at pH 6.0.

Under the molecular crowding condition of 40 wt% PEG 200, the stability of triplexes was also measured, which is shown in Figure 4. As demonstrated in Figure 4a, in 40 wt% PEG 200, all three of the intramolecular triplexes had only one melting stage, and the *T*_m_ values for Intra-CG2, Intra-CG4, and Intra-CG6 were 47.3, 58.0, and 65.3 °C, respectively, which are slightly decreased compared with those in the absence of a crowding agent. These results are consistent with our previous report [9]. On the other hand, the melting curves of intermolecular triplexes did not show two transitions clearly (Figure 4b). Only Hp-CG2+T-CG2 still displayed two stages of melting, which were hard to distinguish. The *T*_m_ values for Hp-CG2+T-CG2 of Hoogsteen base pairs and Watson–Crick base pairs in 40 wt% PEG 200 were 31.8 and 49.3 °C, respectively. Comparing with the *T*_m_s of Hoogsteen and Watson–Crick in the absence of PEG 200, which were 20.5 and 65.5 °C respectively, the results indicate that Hoogsteen base pairs were stabilized substantially by molecular crowding while Watson-Crick base pairs were destabilized. This corresponds well with the conclusions from our previous work [9]. Both Hp-CG4+T-CG4 and Hp-CG6+T-CG6 shifted to a one stage melting curve, like those of the intramolecular triplexes, showing a single melting temperature of 50.0 and 55.0 °C, respectively. In addition, similar to the results seen in the absence of PEG 200, higher C^+^_*_G-C content resulted in a higher triplex melting temperature under molecular crowding conditions. The increment of *T*_m_ between Intra-CG4 and Intra-CG6 was 7.3 °C, which were higher than 6.1 °C as seen in the absence of crowding agents. This suggests that the effect of C^+^_*_G-C on triplex stability is more obvious under molecular crowding conditions.

### 2.2. The Effects of K^+^ on the Stability of Triplexes in the Absence of PEG 200

**Figure 5:**
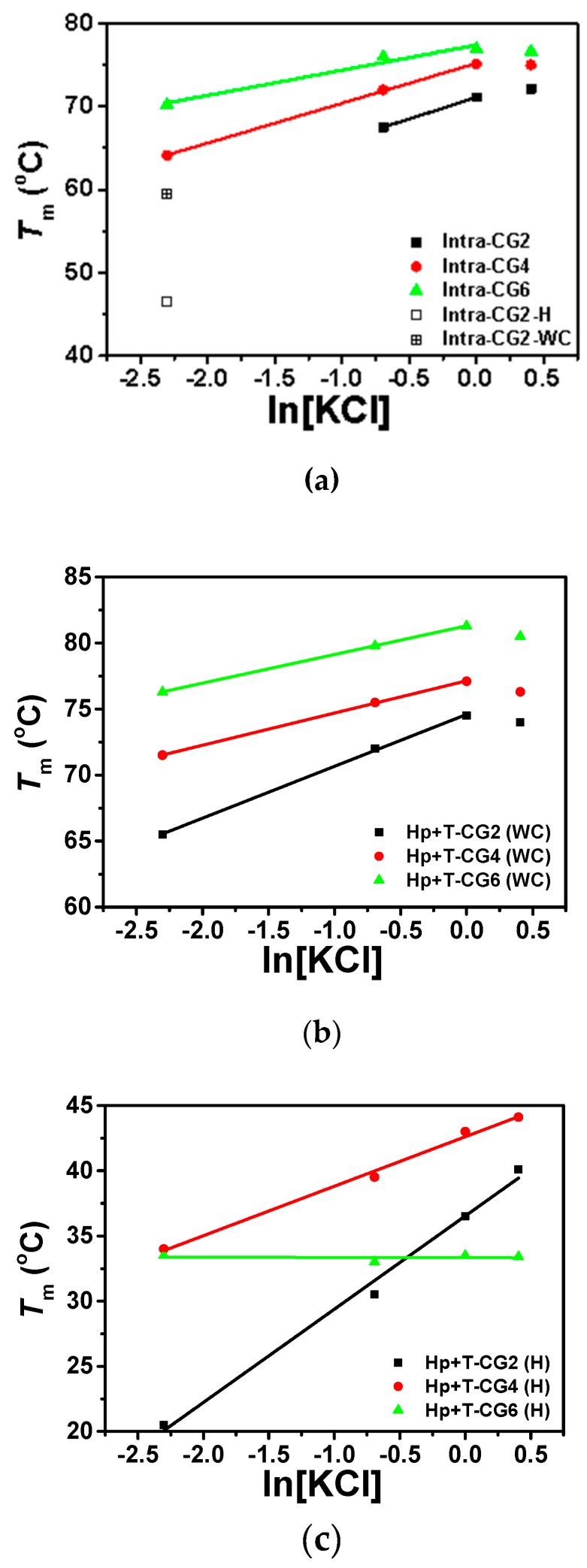
The plot of *T*_m_ vs. ln[KCl] of (**a**) intramolecular triplexes (*T*_m_ of Intra-CG2 in 0.1 M KCl was separately plotted with Hoogsteen base pairs and Watson–Crick base pairs displayed as open square and + open square, respectively), (**b**) Watson–Crick base pairs of intermolecular triplexes, and (**c**) Hoogsteen base pairs of intermolecular triplexes in the buffer containing 50 mM MES at pH 6.0.

The stability of intramolecular and intermolecular triplexes in the absence of PEG 200 with different concentrations of KCl were measured by UV absorption, and the results are shown in Figure 5, Figures S2 and S3, Table 2.

For intramolecular triplexes Intra-CG2, Intra-CG4, and Intra-CG6, as shown in Figures S2 and S3, most of them only showed one melting stage in their melting curves, while Intra-CG2 had two close melting transitions under low KCl conditions ([KCl] ≤ 100 mM). As listed in Table 2, triplex with higher C^+^_*_G-C content had a higher melting temperature under these conditions with the same KCl concentration. To further analyze the relationship between intramolecular triplex stability and potassium concentration, *T*_m_s vs. natural logarithm of KCl concentration were plotted in Figure 5a. The melting temperatures of all three intramolecular triplexes were enhanced with increase in K^+^ concentration when the K^+^ concentration was below 1.0 M. The results indicate that the presence of potassium can stabilize the formation of triplex at low concentrations. The most likely reason for this stabilization is that the positively charged K^+^ bind to the negatively charged phosphate backbones present in nucleic acid strands, reducing the repulsion between nucleic acid strands. There is a linear relationship between *T*_m_ values and ln[KCl] at concentrations between 0.1 M and 1.0 M as shown in Figure 5a, and the values for the slopes of d*T*_m_/dln[KCl] of Intra-CG2, Intra-CG4, and Intra-CG6 were 5.91, 4.80, and 3.03, respectively. A smaller slope was demonstrated in the sequence with higher C^+^_*_G-C content, suggesting that fewer K^+^ were bound to the hairpin structure in high C^+^_*_G-C content triplex. The reason for this might be that higher C^+^_*_G-C content in acidic conditions resulted in more protonated cytosines, causing repulsion of positively charged K^+^. C^+^_*_G-C content showed more obvious effects on the stability of triplexes at high potassium concentrations. For Intra-CG2, there were only two C^+^_*_G-C triplets formed, and Δ*T*_m_ of Intra-CG2 between 1.5 M KCl and 1.0 M KCl was 1.0 °C, suggesting a slightly increasing stability. For Intra-CG4, the Δ*T*_m_ between 1.5 M KCl and 1.0 M KCl was −0.1 °C, indicating that the stability of Intra-CG4 did not change when the concentration of KCl was increased from 1.0 M to 1.5 M. For Intra-CG6, there were six C^+^_*_G-C triplets in the triplex structure. The increase in the melting temperature gradually slowed as KCl concentration increased, and Δ*T*_m_ between 1.5 M KCl and 1.0 M KCl was −0.3 °C, suggesting no significant change in the stability of triplex. The stability of triplexes and duplexes decreased at high salt concentrations due to the non-specific binding of cations to the bases in single strands, although cations generally bind to phosphate ions at lower salt concentrations.

For intermolecular triplexes Hp-CG2+T-CG2, Hp-CG4+T-CG4, and Hp-CG6+T-CG6, the unwinding of single strands and hairpin structures occurred independently in the absence of crowding agent, so there were two melting state transitions (Figure S3). This did not alter even under high potassium ion conditions. As mentioned above, higher melting stages were related to the unwinding of Watson-Crick base pairs, and the lower stages were associated with the unwinding of Hoogsteen base pairs. The two *T*_m_ values are listed in Table 2 and were plotted against the natural logarithm of KCl concentration in Figure 5b,c, respectively. For Watson-Crick base pairs (Figure 5b), *T*_m_s of all three intermolecular triplexes were enhanced with increase in KCl concentrations in the 0.1 M to 1.0 M range, showing a linear relationship. Similar to the results of intramolecular triplexes, the fitted slopes (d*T*_m_/dln[KCl]) of Watson-Crick base pairs of Hp-CG2+T-CG2, Hp-CG4+T-CG4, and Hp-CG6+T-CG6 were 3.93, 2.44, and 2.17, respectively, demonstrating a smaller slope in a sequence with higher C^+^_*_G-C content. In addition, at 1.5 M KCl concentration, all the three *T*_m_s of Watson-Crick base pairs in intermolecular triplexes decreased, indicating destabilization of hairpin structures in triplexes at a high concentration of potassium.

On the other hand, *T*_m_s of Hoogsteen base pairs in Hp-CG2+T-CG2, Hp-CG4+T-CG4, and Hp-CG6+T-CG6 showed different results (Figure 5c). For Hp-CG2+T-CG2 and Hp-CG4+T-CG4, *T*_m_s of Hoogsteen base pairs increased with increasing potassium concentrations, including a high concentration of 1.5 M. *T*_m_ of Hp-CG4+T-CG4 was higher than Hp-CG2+T-CG2 regardless of the conditions used, and the increment observed in Hp-CG4+T-CG4 was smaller than that in Hp-CG6+T-CG6, which was again similar to the results seen with Watson-Crick base pairs. However, Hp-CG6+T-CG6 was unique in the fact that *T*_m_ of its Hoogsteen base pairs was lower than that of Hp-CG4+T-CG4 in every condition studied, and there was almost no change in *T*_m_ of its Hoogsteen base pairs when the concentration of KCl was increased. The most likely explanation for this is the formation of intermolecular G-quartets in the presence of K^+^ due to the high C^+^_*_G-C content in Hp-CG6. The fitted slopes (d*T*_m_/dln[KCl]) of Hoogsteen base pairs of Hp-CG2+T-CG2, Hp-CG4+T-CG4, and Hp-CG6+T-CG6 were 7.15, 3.80, and −0.02, respectively. This result is similar as observed with Watson–Crick base pairs in that the slope decreased when increasing the content of C^+^_*_G-C.

### 2.3. The Effect of K^+^ on the Stability of Triplexes in the Presence of PEG 200

**Figure 6:**
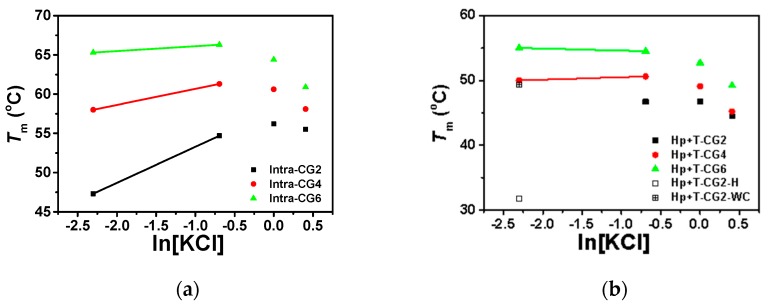
The plot of *T*_m_ vs. ln[KCl] of (**a**) intramolecular triplexes and (**b**) intermolecular triplexes in the buffer containing 50 mM MES and 40 wt% PEG 200 at pH 6.0. *T*_m_ of Hp-CG2+T-CG2 in 0.1 M KCl was separately plotted with Hoogsteen base pairs and Watson–Crick base pairs displayed with open square and + open square, respectively.

We utilized 40 wt% PEG 200 to mimic molecular crowding conditions in cells. The effects of K^+^ on the stability of triplexes were further characterized by UV melting curves. The results are shown in Table 3 and Figure 6, Figures S4 and S5.

Based on our previous work [6], under molecular crowding conditions, Watson–Crick base pairs were destabilized while Hoogsteen base pairs were stabilized. As a result, both intramolecular and intermolecular triplexes showed only one stage of melting (Figures S4 and S5), except in Hp-CG2+T-CG2 at low KCl concentrations ([KCl] ≤ 0.1 M). Typically, the melting temperatures of intermolecular triplexes are lower than intramolecular triplexes in the presence of potassium. Both intramolecular and intermolecular triplexes showed similar trends in the effects of potassium concentration on triplex stability. As shown in Figure 6a,b, under molecular crowding conditions with low concentrations of KCl from 0 M to 0.1 M, *T*_m_s of all triplexes increased with increasing potassium concentrations, except for Hp-CG2+T-CG6. Interestingly, the slopes of intramolecular triplexes Intra-CG2, Intra-CG4, and Intra-CG6, between 0.1 M and 0.5 M K^+^ concentrations, were 4.60, 2.05, and 0.62, respectively, and the slopes of intermolecular triplexes Hp-CG4+T-CG4 and Hp-CG6+T-CG6 were 0.37 and −0.31, respectively. These results suggest that the stabilization of triplex by K^+^ was greatly influenced by the C^+^_*_G-C content. With increasing KCl concentrations, this increment gradually decreased and did not show a linear relationship between *T*_m_ and ln[KCl], as displayed in the absence of crowding agents. At high concentrations of KCl between 1.0 M and 1.5 M, *T*_m_s of all triplexes were decreased with increasing potassium concentrations, suggesting an inhibition of triplex formation by K^+^ under molecular crowding conditions. In addition, Δ*T*_m_s of intramolecular triplexes (Intra-CG2, Intra-CG4, and Intra-CG6) between 1.0 M and 1.5 M KCl concentrations (Δ*T*_m_ = *T*_m_(1.5 M) − *T*_m_(1.0 M)) were −0.7, −2.5, and −3.5 °C, respectively, while Δ*T*_m_s of intermolecular triplexes (Hp-CG2+T-CG2, Hp-CG4+T-CG4, and Hp-CG6+T-CG6) between 1.0 M and 1.5 M KCl concentrations were −2.3, −3.9, and −3.5 °C, respectively. This indicates that the inhibition of potassium was more effective for intermolecular triplexes.

**Figure 7:**
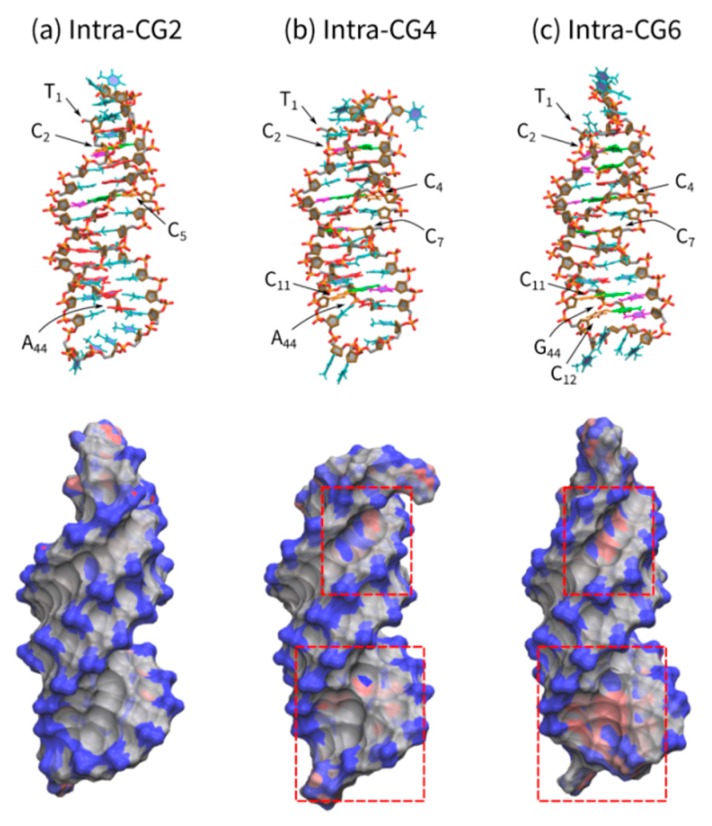
Triplex structures of (**a**) Intra-CG2, (**b**) Intra-CG4, and (**c**) Intra-CG6 obtained via molecular mechanics. All cytosines in the third strand are protonated. Figures in the top panel are triplex structures drawn with a stick model. Red, magenta, orange, green, and cyan sticks in the middle of the structures represent adenine, cytosine, protonated cytosine, guanine, and thymine, respectively. Orange and red sticks at the sides are phosphorus and oxygen atoms, respectively. The bottom panel figures show charge distributions. Blue, gray, and red surfaces show negative, neutral, and positive charges, respectively. The areas with a strong positive charge are highlighted with squares (red dashed line).

We analyzed the charge balance of each triplex with differing C^+^_*_G-C content by molecular dynamics calculations. Figure 7 shows the triplex structures of Intra-CG2, Intra-CG4, and Intra-CG6 obtained via a molecular mechanics method under the assumption that all cytosines in the third strand are protonated. The top panel figures show the triplexes as stick models, and the bottom panel figures show the charge distribution of triplexes. The negative charges on the phosphate groups were scattered along the backbone of all triplexes (blue regions, Figure 7a–c, bottom panel). Conversely, protonated cytosines were positively charged (red regions). In particular, the areas with strong positive charges are highlighted with red squares. Interestingly, the negative charges of Intra-GC4 and Intra-GC6 triplexes were partially canceled by C^+^. These results indicate that cytosine protonation diminished the overall negative charge of triplexes. Such changes in charge balance can interfere with cation binding during triplex formation.

## 3. Discussion

The dramatically different behavior of nucleic acids under molecular crowding conditions suggests that the non-canonical structures of nucleic acids in cellular environment play important roles in gene expression and regulation. The stabilization of Hoogsteen base pairs and the destabilization of Watson-Crick base pairs under molecular crowding conditions might reverse the stability of typical duplex and non-canonical structures [6], which regulate the transcription and translation processes [4]. Cation concentration is also a crucial component of the cellular environment, and many pathological studies have mentioned that significant changes in cation concentration, such as changes in sodium and potassium, occur in diseased cells [10]. The effect of cations on the structural stability of nucleic acids under molecular crowding conditions might be a key factor in the abnormal expression process. In this work, the triplex structure, which has been studied to be abundant in the promoter region and takes part in the regulation of transcription [15], was chosen as our target to explore the differential effects of potassium ion concentration on structural stability in the absence and presence of crowding agents.

In the absence of PEG 200, the stability of triplexes, especially intramolecular triplexes, was greatly affected by the concentration of K^+^. Considering the negatively charged phosphate backbone, the presence of positively charged cations can bind with nucleic acid strands to a large extent and decrease the repulsion of DNA strands. These interactions greatly stabilized both Watson-Crick and Hoogsteen base pairs and resulted in a linear relationship between *T*_m_ and ln[KCl] in the KCl concentration range between 0 to 1.0 M. This suggests that electrostatic interactions are the primary factors that determine the stability of triplex.

On the other hand, in the presence of PEG 200, the effect of K^+^ showed a different trend. Consistent with our previous results [9], under molecular crowding conditions, Hoogsteen base pairs were greatly stabilized, while Watson-Crick base pairs were destabilized. The stabilization of triplexes was greatly reduced by K^+^. Particularly, for the triplex with high C^+^_*_G-C content, the presence of K^+^ made them unstable. The explanation for this might be that crowding conditions have a lower dielectric constant and decrease the electrostatic repulsion of negatively charged DNA strands. The presence of potassium might induce the formation of intermolecular G-quartet and destabilize triplex structures.

Interestingly, the effect of C^+^_*_G-C content on the stability of triplexes was more obvious under molecular crowding conditions. Taking the intramolecular triplexes Intra-CG2, Intra-CG4, and Intra-CG6 as examples, in the absence of K^+^, the Δ*T*_m_s between Intra-CG4 and Intra-CG2 and between Intra-CG6 and Intra-CG4 were 13.5 °C and 9.9 °C, respectively, which is higher than the 10.0 °C and 9.0 °C, respectively, seen in the absence of crowding agents. Similar results were observed in comparing the conditions of 0.1, 0.5, and 1.0 M KCl in the absence and presence of PEG 200. These results suggest that regardless of potassium concentration, C^+^_*_G-C content has a greater impact on triplex stability under molecular crowding conditions. The reason for this might be due to cytosine being easily protonated under molecular crowding conditions.

In our previous work, a temporary intermolecular triplex formation was shown to unwind the G-quadruplex structure in its template strand during transcription, which induces transcription arrest [16]. It might help to reform the duplex structure between template and non-template strands to restart transcription and produce full-length transcripts. Based on our results in this work, the intermolecular triplexes formed more easily under molecular crowding conditions than in dilute conditions, which suggest that triplexes might also play an important role in gene expression and regulation. The differential effects of K^+^ under molecular crowding conditions and dilute conditions shown in this work might provide further information and evidence in the study of triplex function in gene expression and regulation.

## 4. Materials and Methods

### 4.1. Chemicals and Materials

All the oligodeoxynucleotides in this work were purchased from Japan Bio Services Co. (Saitama, Japan) and were used without further purification. The sequences for triplex formation are listed in Table 1. All the DNA samples were dissolved in doubly distilled water purified by a Milli Q system. The concentrations of DNA were determined by absorbance at 260 nm at 90 °C.

To understand the molecular crowding effect on the triplex, we used solution containing 40 wt% PEG200 at different concentration of KCl because, different concentrations of PEG have been used to mimic cellular conditions as a typical crowding agent. 40 wt% in this study is a common upper limit for PEG 200 in mimicking cellular conditions. Usually, the physiological concentration of KCl in cells is close to 0.1 M, which we used for studies in this manuscript. In addition, it is difficult to form triplexes with C^+^G-C triplets in physiological pH, therefore we chose pH 6.0 for our experimental buffers to investigate the effects of molecular crowding and K^+^ concentrations. Previously, our group and others have reported that the effect of molecular crowding on triplex formation at pH 7.0 buffer with different concentration of co-solutes of PEG, EG, 1,2-dimethoxyethane, 1,3-propandiol, 2-methoxyethanol, and glycerol. l. These results showed that all the co-solutes increased *T*_m_ values for Hoogsteen base pairs with increasing with co-solute concentrations [9]. Thus, our experimental condition is useful to understand typical crowding effects on triplex structure stability.

### 4.2. CD Measurement

CD spectra were measured on a JASCO J-820 spectropolarimeter (Hachioji, Tokyo, Japan). The concentrations of all DNA strands were 20 μM and were dissolved in a buffer containing 50 mM 2-morpholino-ethanesulfonic acid (MES) at pH 6.0 with 100 mM KCl in the absence and presence of 40 wt% of PEG 200. Samples were placed at 90 °C for 3 min and then cooled to 4 °C at a rate of −2 °C·min^−1^ for triplex formation before the CD measurement. CD spectra were recorded from 200 to 350 nm at a scanning speed of 100 nm·min^−1^.

### 4.3. UV Melting Assay

UV-Vis absorption spectra were recorded by a Shimadzu 1700 spectrophotometer (Kyoto, Kyoto, Japan) connected to a thermoprogrammer. 20 μM DNA samples were prepared in a buffer containing 50 mM MES with 0, 100, 500, 1000, 1500 mM KCl in the absence and presence of 40 wt% PEG 200 at pH 6.0. After PEG 200 was added, pH of the buffers were regulated to 6.0, and the concentration of KCl did not affect pH of the solution. We performed UV melting experiments with 20 μM of DNA with 1 or 0.1 cm cell to keep the absorption in a range from 0 to 1.0. After incubating at 90 °C for 3 min, DNA samples were annealed by cooling from 90 °C to 0 °C at a rate of −2 °C·min^−1^ for triplex formation. The stabilities of triplex DNA were characterized by the changes in UV melting temperatures. A heating rate of 0.5 °C·min^−1^ was applied in the melting process of DNA samples from 0 °C to 90 °C. The values of *T*_m_ were obtained from the peaks of dA/dT plot for the melting curves, and the errors of analysis were ±1 °C.

### 4.4. Molecular Mechanics Studies

The structures of Intra-CG2, Intra-CG4, and Intra-CG6 were prepared using molecular mechanics. These initial structures were built by Discovery Studio [17]. Potassium cations were placed around phosphate groups of DNAs to neutralize the systems. The system sizes for these structures were set to 10.580 nm × 10.580 nm × 10.580 nm, where the distance between DNA and edge of the system was at least 2.0 nm. The systems were filled with water molecules. A force field of ff14SB [18] was applied to DNA and cations; and TIP3P [19] was applied to water molecules. We adopted General AMBER force field (GAFF) utilizing protonated cytosine, which was generated using the Antechamber and Residuegen modules included in AMBERTools [20,21]. The partial atomic charges of protonated cytosine were determined by a restrained electrostatic potential (RESP) calculation using Gaussian09 software [22]. These force fields were converted by the ACPYPE program [23]. These structures were optimized using the MD simulation package Gromacs ver. 5.1.2 [24]. After water molecules and cations were optimized by 10,000 steps, DNAs were optimized by 10,000 steps.

## 5. Conclusions

The stability of the three intramolecular and three intermolecular triplex-forming sequences of different C^+^_*_G-C content was measured in the absence and presence of a crowding agent with different concentrations of K^+^. Both in the absence and presence of the crowding agent PEG, the formation of triplexes was stabilized at low concentrations of KCl. For triplexes with higher C^+^_*_G-C content, the effect of KCl on triplex stabilization was weaker. Under molecular crowding conditions, Watson-Crick base pairs and Hoogsteen base pairs were destabilized and stabilized, respectively. Interestingly, with lower KCl concentrations (≤1.0 M), the decrease of the triplex stability by KCl concentration reduction was significantly smaller than that in dilute aqueous solutions. Importantly, destabilization of all six triplexes by K^+^ were observed in the range of 1.0 M to 1.5 M under molecular crowding conditions. In addition, C^+^_*_G-C content had greater influence on the stability of triplexes under molecular crowding conditions.

The quantitative information about the effects of potassium concentration on triplex stability under molecular crowding conditions might help further our understanding of the function and regulation of triplexes in bioprocesses.

## Figures and Tables

**Table 1:** Sequences used for triplex formation in this work.

Name	Sequences (5′-3′)
Intra-CG2	TCTTCTTTTTTTTTTTTTTTTTTCTTCTTTTTAGAAGAAAAAAA
Intra-CG4	TCTTCTCTTTCTTTTTTCTTTCTCTTCTTTTTAGAAGAGAAAGA
Intra-CG6	TCCTCTCTTTCCTTTTCCTTTCTCTCCTTTTTAGGAGAGAAAGG
Hp-CG2	TTTTTTTCTTCTTTTTAGAAGAAAAAAA
T-CG2	TCTTCTTTTTTT
Hp-CG4	TCTTTCTCTTCTTTTTAGAAGAGAAAGA
T-CG4	TCTTCTCTTTCT
Hp-CG6	CCTTTCTCTCCTTTTTAGGAGAGAAAGG
T-CG6	TCCTCTCTTTCC

**Table 2:** The melting temperature of 20 μM intramolecular and intermolecular triplexes in the absence of PEG 200 with 0, 0.1, 0.5, 1.0, and 1.5 M KCl.

*T*_m_ (°C)	KCl (M)									
	0		0.1		0.5		1.0		1.5	
	H ^1^	WC ^2^	H ^1^	WC ^2^	H ^1^	WC ^2^	H ^1^	WC ^2^	H ^1^	WC ^2^
Intra-CG2	31.5	45.9	46.5	59.5	67.5		71.1		72.1	
Intra-CG4	53.1		64.1		72.0		75.1		75.0	
Intra-CG6	62.1		70.2		76.0		76.9		76.6	
Hp-CG2+T-CG2	14.2	59.6	20.5	65.5	30.5	72.0	36.5	74.5	40.1	74.0
Hp-CG4+T-CG4	29.7	65.2	34.0	71.5	39.5	75.5	43.0	77.1	44.1	76.3
Hp-CG6+T-CG6	32.1	71.7	33.5	76.3	33.0	79.8	33.5	81.3	33.4	80.5

^1^ H: Hoogsteen base pair. ^2^ WC: Watson–Crick base pair.

**Table 3:** Melting temperatures of 20 μM intramolecular and intermolecular triplexes in the presence of 40 wt% PEG 200 with 0, 0.1, 0.5, 1.0, and 1.5 M KCl.

*T*_m_ (°C)	KCl (M)									
	0		0.1		0.5		1.0		1.5	
	H ^1^	WC ^2^	H ^1^	WC ^2^	H ^1^	WC ^2^	H ^1^	WC ^2^	H ^1^	WC ^2^
Intra-CG2	36.7		47.3		54.7		56.2		55.5	
Intra-CG4	50.2		58.0		61.3		60.6		58.1	
Intra-CG6	60.1		65.3		66.3		64.4		60.9	
Hp-CG2+T-CG2	25.5	45.6	31.8	49.3	46.7		46.8		44.5	
Hp-CG4+T-CG4	47.8		50.0		50.6		49.1		45.2	
Hp-CG6+T-CG6	53.3		55.0		54.5		52.7		49.2	

^1^ H: Hoogsteen base pair. ^2^ WC: Watson–Crick base pair.

## Supplementary Materials

The following are available online, Figure S1: The normalized UV melting curves of Intra-CG2, Intra-CG4, Intra-CG6, Hp-CG2+T-CG2, Hp-CG4+T-CG4, and Hp-CG6+T-CG6 at 295 nm in a buffer containing 50 mM 2-morpholino-ethanesulfonic acid (MES) at pH 6.0 in the absence of PEG 200 and K^+^. Figure S2: (a–c) Melting curves of Intra-CG2, Intra-CG4, and Intra-CG6 at 260 nm in buffers containing 50 mM MES (pH 6.0 at 25 °C) and 0, 0.1, 0.5, 1.0, and 1.5 M KCl in the absence of crowding agents. (d–f) Melting curves of Intra-CG2, Intra-CG4, and Intra-CG6 at 295 nm in buffers containing 50 mM MES (pH 6.0 at 25 °C) and 0, 0.1, 0.5, 1.0, and 1.5 M KCl in the absence of crowding agents. Figure S3: Melting curves of (a–c) Hp-CG2+T-CG2, Hp-CG4+T-CG4, and Hp-CG6+T-CG6 at 260 nm in buffers containing 50 mM MES (pH 6.0 at 25 °C) and 0, 0.1, 0.5, 1.0, and 1.5 M KCl in the absence of crowding agents, (d–f) Hp-CG2+T-CG2, Hp-CG4+T-CG4, and Hp-CG6+T-CG6 at 295 nm in buffers containing 50 mM MES (pH 6.0 at 25 °C) and 0, 0.1, 0.5, 1.0, and 1.5 M KCl in the absence of crowding agents. Figure S4: (a–c) Melting curves of Intra-CG2, Intra-CG4, and Intra-CG6 at 260 nm in the buffers containing 50 mM MES (pH 6.0 at 25 °C) and 0, 0.1, 0.5, 1.0, and 1.5 M KCl in the presence of 40 wt% PEG 200. (d–f) Melting curves of Intra-CG2, Intra-CG4, and Intra-CG6 at 295 nm in the buffers containing 50 mM MES (pH 6.0 at 25 °C) and 0, 0.1, 0.5, 1.0, and 1.5 M KCl in the presence of 40 wt% PEG 200. Figure S5: (a–c) Melting curves of Hp-CG2+T-CG2, Hp-CG4+T-CG4, and Hp-CG6+T-CG6 at 260 nm in buffers containing 50 mM MES (pH 6.0 at 25 °C) and 0, 0.1, 0.5, 1.0, and 1.5 M KCl in the presence of 40 wt% PEG 200. (d–f) Melting curves of Hp-CG2+T-CG2, Hp-CG4+T-CG4, and Hp-CG6+T-CG6 at 295 nm in buffers containing 50 mM MES (pH 6.0 at 25 °C) and 0, 0.1, 0.5, 1.0, and 1.5 M KCl in the presence of 40 wt% PEG 200.
