# Supplementary material for: Effect of Potassium Concentration on Triplex Stability under Molecular Crowding Conditions
Source: Molecules. 2020 Jan 17;25(2):387. doi: 10.3390/molecules25020387 (PMC7024179; doi:10.3390/molecules25020387)
Supplement: Supplementary file 1 [file molecules-25-00387-s001.pdf]

Supporting Information

Effect of potassium concentration on triplex stability under molecular crowding conditions

Ye Teng [1, 2], Hisae Tateishi-Karimata [1], Tatsuya Ohyama [1], and Naoki Sugimoto [1, 3] *

[1] Frontier Institute for Biomolecular Engineering Research (FIBER), Konan University, 7-1-20 Minatojima-Minamimachi, Chuo-ku, Kobe 650-0047 (Japan);

[2] School of Pharmacy, Changchun University of Chinese Medicine, 1035 Boshuo Road, Changchun, Jilin 130117 (China);

[3] Graduate School of Frontiers of Innovative Research in Science and Technology (FIRST), Konan University, 7-1-20 Minatojima-Minamimachi, Chuo-ku, Kobe, 650-0047 (Japan).

* Correspondence: sugimoto@konan-u.ac.jp

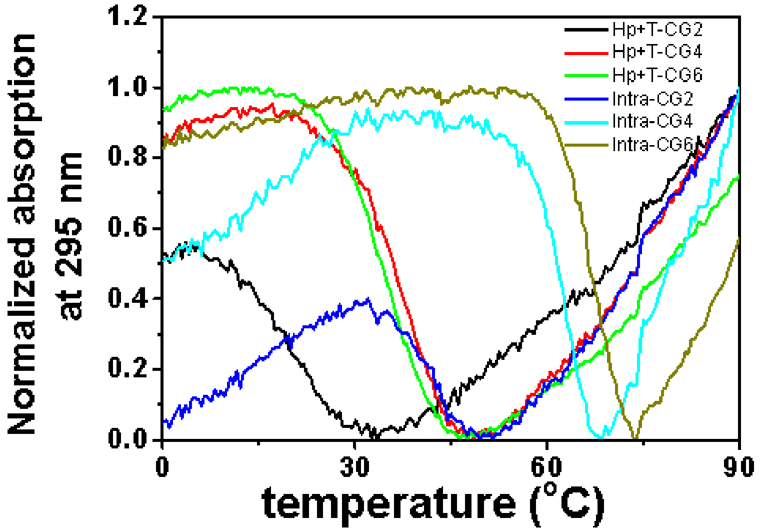

Figure S1. The normalized UV melting curves of 20 μM Intra-CG2, Intra-CG4, Intra-CG6, Hp-CG2+T-CG2, Hp-CG4+T-CG4, and Hp-CG6+T-CG6 at 295 nm in a buffer containing 50 mM 2-morpholino-ethanesulfonic acid (MES) and 0.1 M KCl at pH 6.0 in the absence of PEG 200.

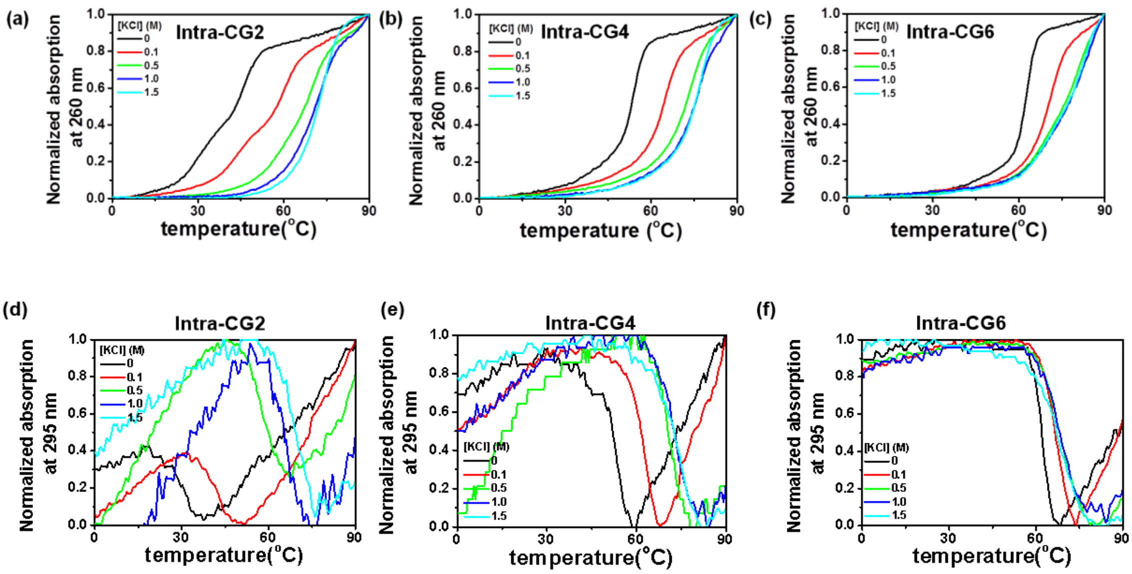

Figure S2. (a)-(c) The melting curves of Intra-CG2, Intra-CG4, and Intra-CG6 at 260 nm in the buffers containing 50 mM MES (pH 6.0 at 25 °C) and 0, 0.1, 0.5, 1.0, and 1.5 M KCl in the absence of crowding agents. (d)-(f) The melting curves of Intra-CG2, Intra-CG4, and Intra-CG6 at 295 nm in the buffers containing 50 mM MES (pH 6.0 at 25 °C) and 0, 0.1, 0.5, 1.0, and 1.5 M KCl in the absence of crowding agents.

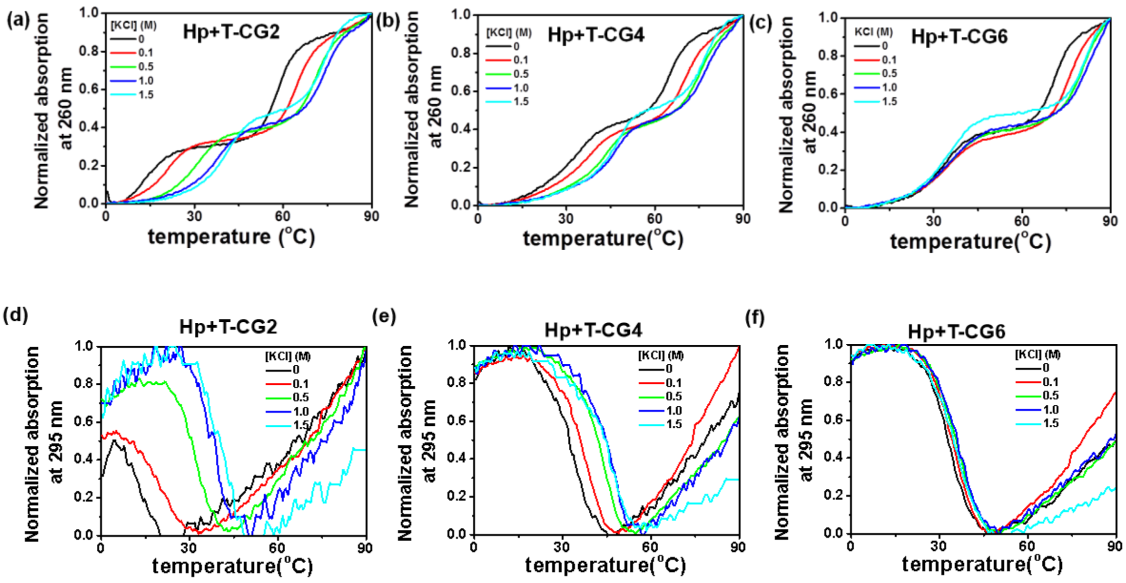

Figure S3. (a)-(c) The melting curves of Hp-CG2+T-CG2, Hp-CG4+T-CG4, and Hp-CG6+T-CG6 at 260 nm in the buffers containing 50 mM MES (pH 6.0 at 25 °C) and 0, 0.1, 0.5, 1.0, and 1.5 M KCl in the absence of crowding agents. (d)-(f) The melting curves of Hp-CG2+T-CG2, Hp-CG4+T-CG4, and Hp-CG6+T-CG6 at 295 nm in the buffers containing 50 mM MES (pH 6.0 at 25 °C) and 0, 0.1, 0.5, 1.0, and 1.5 M KCl in the absence of crowding agents.

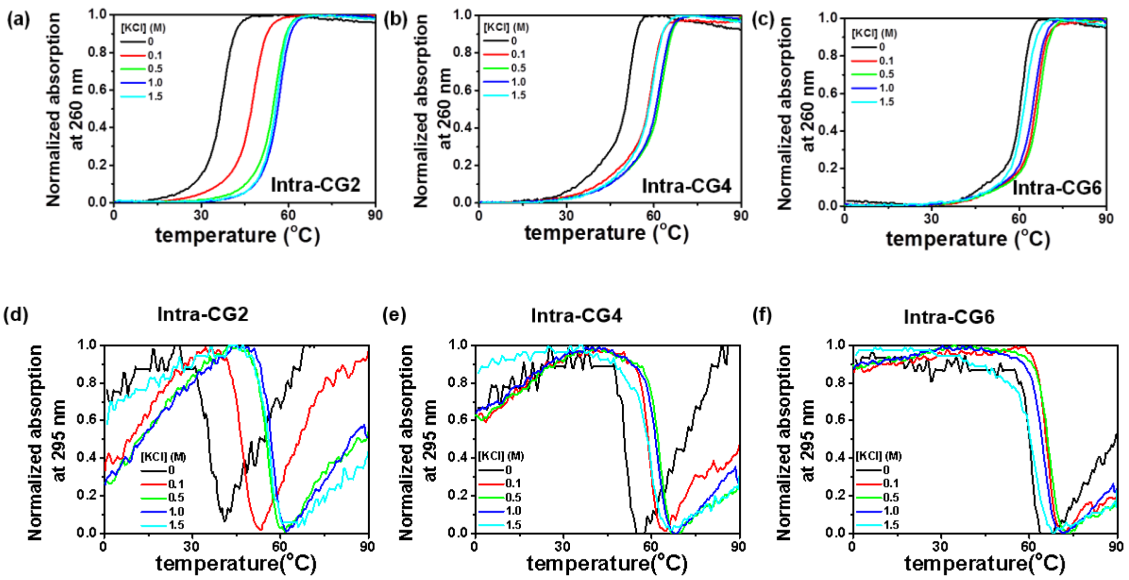

Figure S4. (a)-(c) The melting curves of Intra-CG2, Intra-CG4, and Intra-CG6 at 260 nm in the buffers containing 50 mM MES (pH 6.0 at 25 °C) and 0, 0.1, 0.5, 1.0, and 1.5 M KCl in the presence of 40 wt% PEG 200. (d)-(f) The melting curves of Intra-CG2, Intra-CG4, and Intra-CG6 at 295 nm in the buffers containing 50 mM MES (pH 6.0 at 25 °C) and 0, 0.1, 0.5, 1.0, and 1.5 M KCl in the presence of 40 wt% PEG 200.

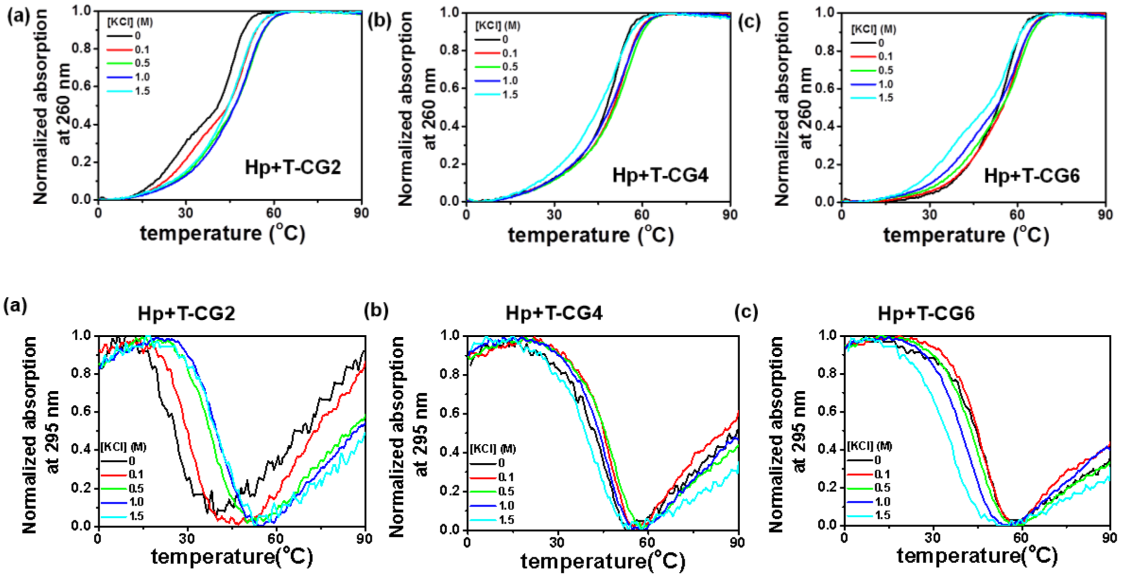

Figure S5. (a)-(c) The melting curves of Hp-CG2+T-CG2, Hp-CG4+T-CG4, and Hp-CG6+T-CG6 at 260 nm in the buffers containing 50 mM MES (pH 6.0 at 25 °C) and 0, 0.1, 0.5, 1.0, and 1.5 M KCl in the presence of 40 wt% PEG 200. (d)-(f) The melting curves of Hp-CG2+T-CG2, Hp-CG4+T-CG4, and Hp-CG6+T-CG6 at 295 nm in the buffers containing 50 mM MES (pH 6.0 at 25 °C) and 0, 0.1, 0.5, 1.0, and 1.5 M KCl in the presence of 40 wt% PEG 200.
